# Antioxidant Activity of Various Parts of *Cinnamomum cassia* Extracted with Different Extraction Methods

**DOI:** 10.3390/molecules17067294

**Published:** 2012-06-13

**Authors:** Cheng-Hong Yang, Rong-Xian Li, Li-Yeh Chuang

**Affiliations:** 1 Department of Electronic Engineering, National Kaohsiung University of Applied Sciences, Kaohsiung 807, Taiwan; 2 Institute of Biotechnology and Chemical Engineering, I-Shou University, Kaohsiung 840, Taiwan

**Keywords:** *Cinnamomum cassia*, antioxidant activity, supercritical fluid extraction, ethanol extraction

## Abstract

The aim of this study was to investigate the antioxidant activities of various parts (barks, buds, and leaves) of *Cinnamomum cassia* extracted with ethanol and supercritical fluid extraction (SFE). For the antioxidant activity comparison, IC_50_ values ofthe SFE and ethanol extracts in the DPPH scavenging assay were 0.562–10.090 mg/mL and 0.072–0.208 mg/mL, and the Trolox equivalent antioxidant capacity (TEAC) values were 6.789–58.335 mmole Trolox/g and 133.039–335.779 mmole Trolox/g, respectively. In addition, the total flavonoid contents were 0.031–1.916 g/ 100 g dry weight of materials (DW) and 2.030–3.348 g/ 100 g DW, and the total phenolic contents were 0.151–2.018 g/ 100 g DW and 6.313–9.534 g/ 100 g DW in the SFE and ethanol extracts, respectively. Based on the results, the ethanol extracts of *Cinnamon* barks have potential value as an antioxidant substitute and this study also provide a better technique to extract the natural antioxidant substances from *C. cassia*.

## 1. Introduction

Many methods for the evaluation of the antioxidant activity of natural product extracts have been developed, such as active oxygen species (e.g., superoxide anion, peroxy radical, and hydroxyl radical) scavenging capability determination, radical (not a natural free radical found in the body) scavenging activity determination, including 1,1-diphyl-2-picrylhydrazyl (DPPH) radical and 2,2'-azinobis-(3-ethyl- benzothiazline)-6-sulfonate radical cation (ABTS^.+^), and enzymatic or nonenzymatic measurements of LPO-inhibiting effects [1].

Antioxidative traditional Chinese medicine (TCM) is an attractive target for the study as a model of antioxidant-based composite therapy against cerebral oxidative damage, as TCM has been used as an alternative medicine for treating complex pathophysiological conditions, even for diseases that Western medicine has failed to allocate a specific diagnostic name.

In view of increasing environmental and health concern about traditional organic solvent extraction, supercritical fluid extraction (SFE) has received much attention in the past several years. The advantages of SFE using carbon dioxide compared with ethanol extraction are that it is relatively rapid and environmentally friendly, such as low operating temperature (hence no thermal degradation of most of the active compounds ocurrs); high selectivity in the extraction compounds; no solvent residue. However, the drawback in the use of SFE-CO_2_ is the cost of supplementary equipment and its low polarity, making the extraction of polar components difficult. Even small amounts of polar modifiers, such as methanol or ethanol, can enhance the efficiency of extraction, the polar solvent extraction provide a higher efficiency on the separation of polar active components [2]. The disadvantages of solvent extraction are: low yield, loss of volatile compounds, long extraction time, toxic solvent residues and degradation of active compounds, due to heat.

The main classes of bioactive compounds from plants include flavonoids, terpenes, alkaloids, and coumarins [3]. Favonoids are polyphenolic compounds that produce the flavor of fruits and vegetable as well as the red and blue pigments of plants, and have been used in taxonomic studies of angiosperms. Terpenes are responsible for the fragrance of essential oils, and they consist of repeating isoprene units with monoterpenes having two isoprene units. Coumarins are most abundant in grasses and have been found to have wide-ranging activity, including antimicrobial, antiviral, antithrombotic and anti-inflammatory properties. Alkaloids are compounds that consist of a heterocyclic ring with a nitrogen atom. They include caffeine, morphine, and nicotine.

*Cinnamon* has a long history of use as preservative and for medicinal purposes in the East. In our previous studies, we examined the antibacterial activities of 58 herbal plants extracted by 95% ethanol. The results revealed that *C. cassia* sticks exhibited obvious broad-spectrum antimicrobial activity against clinical drug-resistant *Pseudomonas aeruginosa* isolates [4]. Following the study, we extracted *C. cassia* sticks by supercritical fluid extraction (SFE) under various extraction conditions to determine the best SFE conditions. The extraction conditions were examined in the range of 4,000–6,000 psi and 40–50 °C [2]. The results showed that the highest yield of the antimicrobial substance, (*E*)-cinnamaldehyde, can be isolated at 4,500 psi and 45 °C. In addition to our previous studies, there are many literature reports that barks and leaves of *Cinnamon* present various bioactive functions, such as the anti-diabetic effects presented by *C. cassia* barks [5], the antibacterial and antioxidant activities of *C. zeylanicum* barks and leaves [6], the anti-inflammation and anti-proliferation of tumor cell activities presented by *C. osmophloeum* barks [7]. *C. osmophloeum* leaves have antifungal activities [8]. However, regarding to the antioxidant activities of various parts of *Cinnamon*, not much related research has been reported.

In this study, we aimed to compare the antioxidant activities of extracts of various parts of *C. cassia* (barks, buds and leaves) obtained by supercritical carbon dioxide extraction and ethanol extraction. The total flavonoid and phenolic contents in each extract were also determined. The results of this study not only provided a better technique for bioactive substance extraction from *C. cassia*, but also revealed the best parts of *C. cassia* to isolate the active constituents. 

## 2. Results and Discussion

### 2.1. Extraction Efficiency

The extraction yield is expressed as the percentage of total mass of extracts (*Mext*) with respect to the mass of material loaded onto the apparatus (*Mo*):



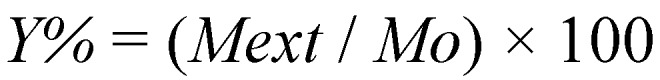



As shown in Table 1, for the ethanol extraction, dried yields of buds, barks and leave were 6.85%, 12.73% and 23.02% (w/w), respectively. For the SFE extraction, the yields of buds were 3.34%, 0.13% and 0.46% at “+10 min”, “−10 min” and “wash” collections, respectively. The yields of leave were 0.68%, 0.36% and 1.74% at “+10 min”, “−10 min” and “wash” collections, respectively. The yields of barks were 0.90%, 0.16% and 0.22% at “+10 min”, “−10 min” and “wash” collections, respectively. Regarding the dry yields, this preliminary experiment revealed that the ethanol extraction obtained higher yield than the supercritical CO_2_ extraction. For the ethanol extraction, the extract yield from leave is higher than the other parts of *C. cassia*. However, the buds showed a highest yield among all of the parts of *C. cassia* by supercritical CO_2_ extraction collected during the first 10 min.

**Table 1:** Total phenolics (TPC) and flavonoids (TFC) in the various extracts of *C. cassia.*

Extracts		Ethanol	SFE	SFE	SFE
			(+10 min)	(−10 min)	(wash)
**Extraction yield**	buds	6.85%	3.34%	0.13%	0.46%
	barks	12.73%	0.90%	0.16%	0.22%
	leaves	23.02%	0.68%	0.36%	1.74%
**TPC**	buds	6.313 ± 0.15	0.151 ± 0.01	0.812 ± 0.01	1.712 ± 0.01
(g GAE/	barks	9.534 ± 0.26	0.398 ± 0.01	2.018 ± 0.04	1.763 ± 0.01
100 g DW)	leaves	8.854 ± 0.35	0.986 ± 0.02	1.248 ± 0.02	1.338 ± 0.03
**TFC**	buds	2.697 ± 0.17	0.395 ± 0.01	1.322 ± 0.03	2.504 ± 0.05
(g Quercetin/	barks	2.030 ± 0.10	0.031 ± 0.01	1.266 ± 0.10	1.373 ± 0.09
100 g DW)	leaves	3.348 ± 0.29	1.288 ± 0.02	1.086 ± 0.05	1.916 ± 0.05

Values are mean of three replicate determinations (n = 3) ± standard deviation.

### 2.2. Total Phenolic Content

The total phenolic content in the various extracts were determined by a spectrometric method, according to the Folin-Ciocalteu phenol method and calculated as gallic acid equivalent (GAE). Phenolics compounds such as flavonoids, phenolic acid and tannins posses diverse biological activities. These activities might be related to their antioxidant activity [9]. The amounts of total phenolics in the various extracts of *C. cassia* are shown in Table 1. The total phenolic content ranged from 6.313 to 9.534 g GAE/100 g DW and 0.151 to 2.08 g GAE/100 g DW for the ethanol and SFE extracts, respectively. The highest phenolic content is the ethanol extracts of barks (9.534 g GAE/100 g DW), followed by the leaves (8.854 g GAE/100 g DW) and buds (6.313 g GAE/100 g DW). The SFE extracts revealed a lower phenolic content than the ethanol extracts. 

The results revealed that the 95% ethanol extracts of leaves total phenolic content (8.854 g/100 g DW) is about two times the total flavonoid content (3.348 g/100 g DW). This result is similar as the literature report [10] that the 50% ethanol extracts of leaves from Chinese *C. cassia* possessed total phenolic contents of 1,558.7 μg/g DW and total flavonoids content of 981.1 μg/g DW. Obviously, the total phenolic content is higher than the flavonoid content in the leaves of *C. cassia*. The total phenolics might be the main substances that contribute the antioxidant activities for *C. cassia*. Comparison of our results with the literature report, show that the 95% ethanol extraction is better than 50% ethanol extraction to obtain a higher content of polyphenolics from *C. cassia*.

### 2.3. Total Flavonoid Content

The total flavonoids, such as flavanols, flavonols, isoflavones, and anthocyanidins, have been reported to have multiple biological effects, including antioxidant activity. The capacity of flvonoids to act as antioxidants depends upon their molecular structure. The position of hydroxyl groups and other features in the chemical structure of flavonoids are important for their antioxidant and free radical scavenging activities. As shown in the Table 1, the total flavonoid content of the ethanol extracts followed the order: leaves (3.348 g/100 g DW) > buds (2.697 g/100 g DW) > barks (2.030 g/100 g DW). Overall, the SFE extracts revealed lower total flavonoid content (0.031–2.504 g/100 g DW) than the ethanol extracts (2.030–3.348 g/100 g DW).

### 2.4. Antioxidant Activities

The principle of antioxidant activity is based on the availability of electrons to neutralize free radicals. In this study, the antioxidant activity of the various extracts from *Cinnamomum cassia* was tested by the DPPH radical scavenging and the Trolox equivalent antioxidant capacity assays.

#### 2.4.1. DPPH Free Radical Scavenging Ability

**Figure 1:**
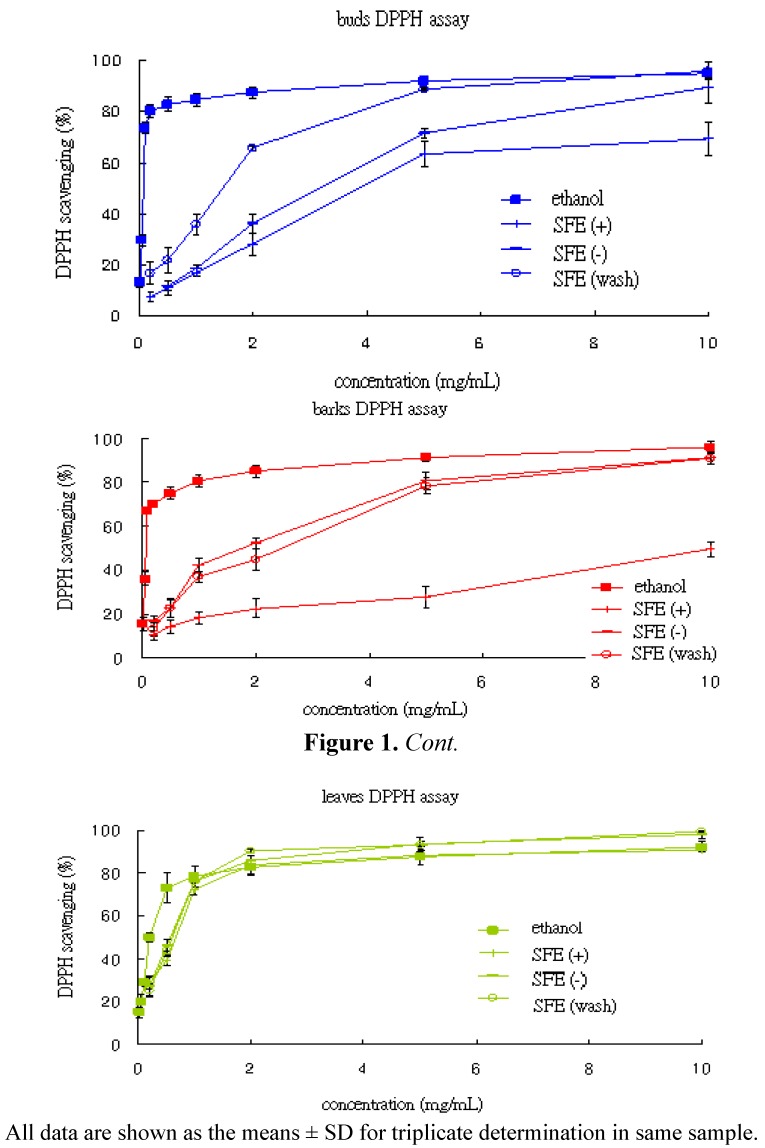
DPPH radical scavenging activity of the various extracts of *C. cassia.*

The effect of antioxidant on DPPH radical scavenging was thought to be due to their hydrogen donating ability or radical scavenging activity. When a solution of DPPH is mixed with a substance that can donate a hydrogen atom, it then leads to a loss of this violet color. Free radical scavenging activities of the various extracts are presented in Table 2. A lower IC_50_ value indicates higher antioxidant activity. The ethanol extracts from leaves, buds and barks of *C. cassia* exhibited remarkable antioxidant activities. The IC_50_ of leaves, buds and barks were 0.208 mg/mL, 0.073 mg/mL and 0.072 mg/mL, respectively. The IC_50_ value of the standard BHT was 0.027 mg/mL. In this study, DPPH radical scavenging activity of test samples increased with increasing its concentration (Figure 1). For the SFE extracts, the “wash” fractions exhibited substantial higher antioxidant activity than the other SFE fractions. Moreover, the SFE extracts from leaves (0.593 mg/mL) showed higher antioxidant activity than the bud (1.457 mg/mL) and bark (2.446 mg/mL) extracts, even the ethanol extracts of leaves possess the lowest antioxidant activity than the other ethanol extracts.

**Table 2:** DPPH and ABTS radical scavenging activity of the various extracts of *C. cassia.*

Extracts		Ethanol	SFE	SFE	SFE
			(+10 min)	(−10 min)	(wash)
**DPPH**	buds	0.073 ± 0.000	3.881 ± 0.366	3.144 ± 0.228	1.457 ± 0.082
IC_50_ (mg/mL)	barks	0.072 ± 0.003	10.090 ± 0.847	1.772 ± 0.221	2.446 ± 0.430
	leaves	0.208 ± 0.022	0.661 ± 0.039	0.562 ± 0.039	0.593 ± 0.038
**TEAC**	buds	133.04 ± 11.32	6.79 ± 1.8	29.10 ± 0.76	58.34 ± 0.64
(mmole trolox/g)	barks	335.78 ± 77.15	10.06 ± 4.3	51.89 ± 13.57	55.67 ± 14.85
	leaves	297.34 ± 65.23	39.74 ± 9.13	43.98 ± 7.06	45.82 ± 8.80

Values are mean of three replicate determinations (n = 3) ± standard deviation.

#### 2.4.2. Trolox Equivalent Antioxidant Capacity (TEAC)

The TEAC assay is one of the most frequent methods for determination of antioxidant activity. It is based on the radical scavenging capacity of antioxidants towards the ABTS radical cation, which is generated by oxidation of ABTS. In ABTS assay, the activity of tested sample extracts are expressed as the minimolar equivalent of Trolox solution, having an antioxidant capacity equivalent to 1 g dry matter of the extract under the experiment investigation. 

**Figure 2:**
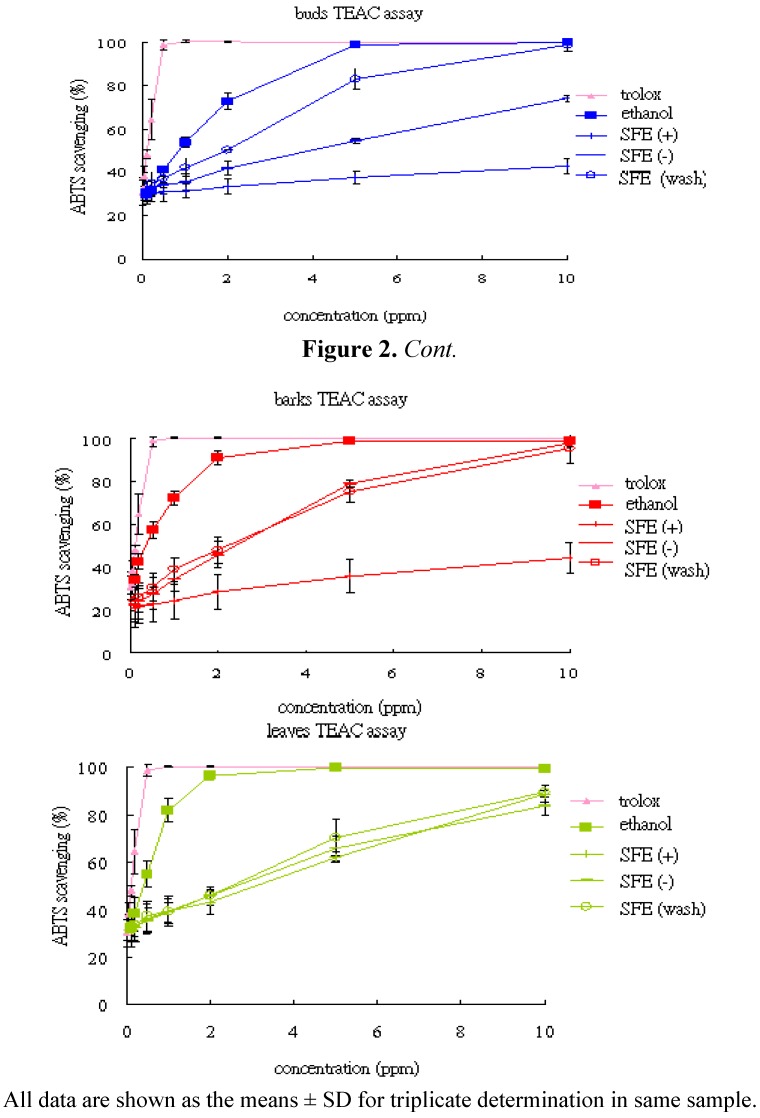
ABTS radical scavenging activity of the various extracts of *C. cassia.*

The efficacy of ABTS cation radical scavenging activity of various extracts of C. cassia (buds, barks and leaves) is shown in Table 2. The antioxidant capacity of the ethanol extracts was 133.04, 335.78, and 297.34 mmol trolox /g for buds, barks and leaves, respectively. In this study, ABTS cation radical scavenging activity of various extracts increased with increasing its concentration (Figure 2). Comparison of the SFE extracts, the extracts obtained from the “washing” fraction ethanol revealed a better ABTS radical scavenging activity than other SFE extracts with 58.34, 55.67 and 45.82 mmol trolox /g for the buds, barks and leaves, respectively. In the present investigation, the ethanol extracts of barks registered the highest TEAC (335 mmol trolox /g), followed by the leave extracts (297 mmol trolox /g) and bud extracts (133 mmol trolox /g). The SFE extracts of the samples showed lower level activity than the ethanol extracts. These results suggested that the active components are substantial higher polarity.

There are significant differences in antioxidant activity among the different extraction methods. This indicated indirectly that the antioxidant activity of the different extracts mainly depended on the content of total phenolic and flavonoid contents. Comparison of the ethanol and SFE extracts showed a significant relationship between the antioxidant capacities and total phenolic contents. The results indicated the major contributors of antioxidant activities of this plant are its phenolics. This is in agreement with previous studies indicating that the phenolic compounds contribute significantly to the antioxidant capacities of Chinese medicine plants [10].

The barks and leaves of cinnamon have been widely studied and reported to possess antibacterial activity [6]. However, the antioxidant activity of various parts of *Cinnamon* extracted with different methods has not been investigated and compared; only the ethanol extracts of *Cinnamon* barks have been evaluated to reveal significant antioxidant activity [11]. The present study compares the bioactivity of various parts from cinnamon and revealed that the ethanol extracts from cinnamon barks had a highest antioxidant activity, with DPPH and ABST radical scavenging capacities of 0.072 mg/mL and 335.78 mg Trolox/g, respectively. The finding is consistent with the previous literature reports by Lin and Lee [11,12]. In addition, the SFE extracts from *Cinnamon* leaves showed higher antioxidant activity than the extracts of *Cinnamon* buds and barks.

Several authors have compared the essential oil obtained by solvent extraction and the product obtained by SFE, they found that solvent extraction obtained higher percentages of terpene hydrocarbons. In contrast, the SFE extracts contained a higher percentage of oxygenated compounds [13]. Regarding the active constituents of the *Cinnamon* extracts, the components obtained from SFE extracts of *Cinnamon* have been determined by GC-MS in our recent study [14]. The major components in the SFE bud extracts were (*E*)-cinnamaldehyde (49–67%), coumarin (4–21%), and O-methoxycinnamaldehyde (4–26%); in the SFE bark extracts they were (*E*)-Cinnamaldehyde (57–69%), coumarin (4–21%), and naphthalene (3–14%); and in the leaf extracts they were eucalyptol (17–24%), bornylene (10–17%) and *n*-hexadecanoic acid (7–19%). According to the literature report [18], the polyphenol contents in alcohol extracts of *Cinnamon* barks were rutin (90.0672%), quercetin (0.172%), kaempferol (0.016%), isorhamentin (0.103%) and catechin (1.9%). The SFE extracts contained a higher amount of (*E*)-cinnamaldehyde, and hence possess higher antimicrobial activity than the ethanol extracts.

## 3. Experimental

### 3.1. Collection of Plant Materials

The various parts of *C. cassia* (barks, buds, and leaves) were collected from Vietnam and China, in 2007, and purchased from local folk medicinal dealers in Kaohsiung, Taiwan. The plant materials were authenticated by the Department of Traditional Chinese Medicine, Kaohsiung Medical University, Chung-Ho Memorial Hospital, Taiwan.

### 3.2. Preparation of the Extracts

For the ethanol extraction, the dried and finely ground (≤1 mm) sample was extracted by 95% ethanol as described elsewhere [3]. The ethanol solution was vacuum dried to obtain the crude extract and stored at 4 °C for the bioactivity assay. For the supercritical fluid extraction, the dried and finely ground (1–2 mm) sample was extracted in a high-pressure pilot unit (NATEX, MIRDC, Kaohsiung, Taiwan) with a 5 L volume extractor vessel, which was filled with the ground barks, buds and leaves (about 2.0 kg, 2.0 kg and 1.4 kg, respectively). The extraction condition, 45 °C and 600–650 bar, were based on our previous studies [2], and the CO_2_ flow rate was about 30 kg CO_2_ h^−1^ kg^−1^ raw materials. The operational parameters set in the separator were 45 bar and 20 °C during the process of extraction. The “+10 min” is defined as the extracts were collected at the first 10 min, “−10 min” is defined as the extracts were collected at 10–20 min, and “wash” is defined as the separator washing ethanol.

### 3.3. Antioxidant Activity Analysis by the DPPH Free Radical Scavenging Method

Antioxidant activity of the dried ethanol extract was measured on the basis of its scavenging activities towards the stable 1,1-diphenyl-2-picrylhydrazyl (DPPH) radical [16]. Briefly, to various concentrations of test samples (1 mL) 0.5 mM DPPH methanol solution (1 mL) was added. After 30 min of incubation in the dark at room temperature, the absorbance was measured against a blank (methanol) at 517 nm using a UV/Visible spectrophotometer. Inhibition of DPPH radical was calculated as a percentage (%) using the formula:



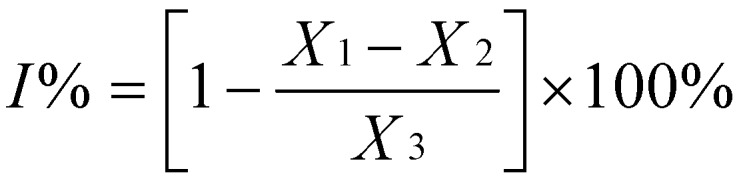



where X_1_ is absorbance of the tested sample (containing tested samples with DPPH methanol solution), X_2_ absorbance of the tested samples without DPPH methanol solution, and X_3_ absorbance of the control (containing all reagents except the tested samples). IC_50_ values (concentration of sample required to scavenge 50% of free radicals) were calculated from the regression equation, prepared from the concentration of the samples and percentage inhibition of free radical formation (percentage inhibition DPPH was assayed). Butylated hydroxytoluene (BHT) was used as a control.

### 3.4. Trolox Equivalent Antioxidant Capacity (TEAC)

The ABTS^.+^radical cation was produced by the method of Miller [17]. A solution of ABTS (10 mg) and potassium peroxodisulfate (2.9 mg) was diluted with 0.01 M pH 7.4 sodium phosphate buffer (10 mL). The mixture was protected from light and stored at room temperature for 12–16 h. Formation of ABTS^.+^ was checked by its absorbance at 734 nm. The ABTS^.+^ solution was diluted with water to an absorbance of 0.80 (±0.05) at 734 nm. For the assays, briefly, samples (0.02 mL) were mixed with ABTS^.+^ solution (1 mL). Reduction of absorbance was measured at 734 nm after 5 min. Trolox was used as the standard for the comparison of antioxidant activity expressed as Trolox equivalent antioxidant capacity (TEAC) by plotting the Trolox calibration curve (from 10 to 300 mg/L) and expressed as milligrams of Trolox equivalents per gram of dried extract. The equation for the Trolox calibration curve was Y = −0.0022·X + 0.7473 (where X = concentration of Trolox equivalents expressed as milligrams of Trolox per gram of dried extract; Y = measured absorbance), and the correlation coefficient was R^2^ = 0.9995.

### 3.5. Antioxidant Activity Analysis by the Total Phenolic Content Determination

The total phenolic content of extracts was determined using the Folin–Ciocalteu reagent [18]. Briefly, each extract (0.2 mL) was shaken for 1 min with 0.5 M Folin-Ciocalteu reagent (1 mL). After 4–8 min mixture was shaken and added 75 g/L sodium carbonate (1 mL), the mixture was shaken once again for 0.5 min. After 2 h, the absorbance was read on the UV/Visible spectrophotometer at 760 nm. The TPC was assessed by plotting the gallic acid calibration curve (from 10 to 200 mg/L) and expressed as milligrams of galic acid equivalents (GAE) per gram of dried extract. The equation for the gallic acid calibration curve was Y = 0.0098·X − 0.0172 (where X = concentration of gallic acid equivalents (GAE) expressed as milligrams of GAE per gram of dried extract; Y = measured absorbance), and the correlation coefficient was R^2^ = 0.9965.

### 3.6. Total Flavonoid Content Determination

The total flavonoid content was established by the reaction with aluminum chloride using the method of Lin [11]. Briefly, each extract (1 mL) was shaken for 1 min and added 10% aluminium chloride (0.1 mL), 1M potassium acetate (0.1 mL) and methanol (3.8 mL). After 40 min at room temperature, the absorbance was read on the UV/Visible spectrophotometer at 415 nm. The flavonoids was assessed by plotting the quercetin calibration curve (from 10 to 200 mg/L) and expressed as milligrams of quercetin equivalents per gram of dried extract. The equation for the quercetin calibration curve was Y = 0.0101X − 0.0632 (where X = concentration of quercetin equivalents expressed as milligrams of quercetin per gram of dried extract; Y = measured absorbance), and the correlation coefficient was R^2^ = 0.9967.

## 4. Conclusions

From the previously motioned results, it can be concluded that the extracts of *Cinnamon* barks exhibited higher antioxidant activity than other parts of cinnamon. This study also demonstrated that the ethanol is the best solvent to obtain the main antioxidant constituents. All these findings indicate that *Cinnamon* extracts possess antioxidant activity. By these findings and purification of the active substance(s) present in the extracts of *Cinnamon*, it will be possible to discover new natural drugs serving as antioxidant agents for application in the nutritional or pharmaceutical fields, in the prevention of free radical-mediated diseases. *Cinnamon* barks could act as a better antioxidant agent than other parts of *Cinnamon* based on their radical scavenging abilities. Further studies are needed to explore the potential phenolic compounds from *Cinnamon* barks and *in vivo* studies are needed for better understanding their mechanism of action.
